# Cerebrovascular Pressure Reactivity According to Long-Pressure Reactivity Index During Spreading Depolarizations in Aneurysmal Subarachnoid Hemorrhage

**DOI:** 10.1007/s12028-022-01669-y

**Published:** 2023-01-25

**Authors:** Renan Sanchez-Porras, Francisco L. Ramírez-Cuapio, Nils Hecht, Martin Seule, Roberto Díaz-Peregrino, Andreas Unterberg, Johannes Woitzik, Jens P. Dreier, Oliver W. Sakowitz, Edgar Santos

**Affiliations:** 1 Department of Neurosurgery, Heidelberg University Hospital, Ruprecht Karls University of Heidelberg, Heidelberg, Germany; 2 Department of Neurosurgery, Berlin Institute of Health, Charité–Universitätsmedizin Berlin, Corporate Member of Freie Universität Berlin, Humboldt-Universität zu Berlin, Berlin, Germany; 3 Center for Stroke Research Berlin, Berlin Institute of Health, Charité–Universitätsmedizin Berlin, Corporate Member of Freie Universität Berlin, Humboldt-Universität zu Berlin, Berlin, Germany; 4 Present Address: Department of Neurosurgery, Kantonsspital St. Gallen, St. Gallen, Switzerland; 5 Present Address: Department of Neurosurgery, Evangelisches Krankenhaus Oldenburg, Carl von Ossietzky University of Oldenburg, Oldenburg, Germany; 6 Department of Neurology, Berlin Institute of Health, Charité–Universitätsmedizin Berlin, Corporate Member of Freie Universität Berlin, Humboldt-Universität zu Berlin, Berlin, Germany; 7 Department of Experimental Neurology, Berlin Institute of Health, Charité–Universitätsmedizin Berlin, Corporate Member of Freie Universität Berlin, Humboldt-Universität zu Berlin, Berlin, Germany; 8 Bernstein Center for Computational Neuroscience Berlin, Berlin, Germany; 9 Einstein Center for Neurosciences Berlin, Berlin, Germany; 10 Present Address: Neurosurgery Center Ludwigsburg-Heilbronn, RKH Klinikum Ludwigsburg, Ludwigsburg, Germany

**Keywords:** Aneurysmal subarachnoid hemorrhage, Cerebrovascular autoregulation, Cerebrovascular reactivity, Long-pressure reactivity index, Spreading depolarization

## Abstract

**Background:**

Spreading depolarization (SD) has been linked to the impairment of neurovascular coupling. However, the association between SD occurrence and cerebrovascular pressure reactivity as a surrogate of cerebral autoregulation (CA) remains unclear. Therefore, we analyzed CA using the long-pressure reactivity index (L-PRx) during SDs in patients with aneurysmal subarachnoid hemorrhage (aSAH).

**Methods:**

A retrospective study of patients with aSAH who were recruited at two centers, Heidelberg (HD) and Berlin (BE), was performed. Continuous monitoring of mean arterial pressure (MAP) and intracranial pressure (ICP) was recorded. ICP was measured using an intraparenchymal probe in HD patients and was measure in BE patients through external ventricular drainage. Electrocorticographic (ECoG) activity was continuously recorded between 3 and 13 days after hemorrhage. Autoregulation according to L-PRx was calculated as a moving linear Pearson’s correlation of 20-min averages of MAP and ICP. For every identified SD, 60-min intervals of L-PRx were averaged, plotted, and analyzed depending on SD occurrence. Random L-PRx recording periods without SDs served as the control.

**Results:**

A total of 19 patients (HD *n* = 14, BE *n* = 5, mean age 50.4 years, 9 female patients) were monitored for a mean duration of 230.4 h (range 96–360, STD ± 69.6 h), during which ECoG recordings revealed a total number of 277 SDs. Of these, 184 represented a single SD, and 93 SDs presented in clusters. In HD patients, mean L-PRx values were 0.12 (95% confidence interval [CI] 0.11–0.13) during SDs and 0.07 (95% CI 0.06–0.08) during control periods (*p* < 0.001). Similarly, in BE patients, a higher L-PRx value of 0.11 (95% CI 0.11–0.12) was detected during SDs than that during control periods (0.08, 95% CI 0.07–0.09; *p* < 0.001). In a more detailed analysis, CA changes registered through an intraparenchymal probe (HD patients) revealed that clustered SD periods were characterized by signs of more severely impaired CA (L-PRx during SD in clusters: 0.23 [95% CI 0.20–0.25]; single SD: 0.09 [95% CI 0.08–0.10]; control periods: 0.07 [95% CI 0.06–0.08]; *p* < 0.001). This group also showed significant increases in ICP during SDs in clusters compared with single SD and control periods.

**Conclusions:**

Neuromonitoring for simultaneous assessment of cerebrovascular pressure reactivity using 20-min averages of MAP and ICP measured by L-PRx during SD events is feasible. SD occurrence was associated with significant increases in L-PRx values indicative of CA disturbances. An impaired CA was found during SD in clusters when using an intraparenchymal probe. This preliminary study validates the use of cerebrovascular reactivity indices to evaluate CA disturbances during SDs. Our results warrant further investigation in larger prospective patient cohorts.

**Supplementary Information:**

The online version contains supplementary material available at 10.1007/s12028-022-01669-y.

## Introduction

To monitor cerebral autoregulation (CA) at the bedside, indices of cerebrovascular reactivity can serve as surrogate markers of CA. One of the most widely investigated cerebrovascular reactivity indices is the pressure reactivity index (PRx), defined as the moving linear Pearson correlation index between mean arterial pressure (MAP) and intracranial pressure (ICP), which correlates with outcome after aneurysmal subarachnoid hemorrhage (aSAH) and traumatic brain injury (TBI) [1]. Recently, the low-frequency sample or long-pressure reactivity index (L-PRx), which correlates well with PRx and is based on longer (1 min) average values of MAP and ICP, has been shown to be similarly associated with outcome after TBI and intracerebral hemorrhage [2–4]. Compared to PRx, however, L-PRx is likely less affected by high-frequency fluctuations and/or noise and can also display pressure reactivity changes that occur during slow MAP waves [5].

Spreading depolarization (SD) describes a propagating wave of neuronal and glial depolarization, near-complete breakdown of the transmembrane neuronal ion gradients, and cytotoxic edema [6]. In DISCHARGE-1 (Depolarisations in Ischaemia After Subarachnoid Haemorrhage-1), a recent prospective, observational, multicenter, cohort, diagnostic phase III trial of 180 patients with severe aSAH, SD variables were included in each multiple regression model for early, delayed, and total brain damage; 7-month outcome; and death. This study concluded that SDs are an independent biomarker of progressive brain injury [7]. In both experimental animal models and patients with aSAH, SD induces tone alterations in resistance vessels, causing either transient vasodilatation and hyperperfusion (physiological hemodynamic response) in normal tissue or initial vasoconstriction and severe hypoperfusion (inverse hemodynamic response = spreading ischemia) in tissue where the neurovascular unit is impaired [8]. Normal and inverse hemodynamic responses to SD also occur after TBI. In TBI, a correlation between inverse hemodynamic responses to SD and CA impairment has been described [9].

In the present study, we monitored CA according to L-PRx in patients with aSAH and investigated the relationship of cerebrovascular reactivity changes with SD occurrence.

## Methods

We conducted a retrospective study of patients with rupture of a saccular aneurysm who underwent surgical treatment. In these patients, electrocorticography (ECoG) and ICP monitoring were simultaneously performed. The study protocol was approved by the Ethics Committee of the University of Heidelberg Medical School (HD) and Charité University Hospital in Berlin (BE). Informed consent was obtained from the patients’ caregivers. Patients were admitted to the neurosurgical intensive care unit (ICU) between February 2005 and July 2010. Included patients presented with World Federation of Neurosurgical Societies scale grades 1–4 and Glasgow Coma Scale scores ≥ 4. The diagnosis was performed by computed tomography angiography or digital subtraction angiography. All patients underwent a craniotomy to execute clip ligation of the aneurysm during the first 72 h after the initial hemorrhage. No patient underwent decompressive craniectomy, so the bone flap was returned and fixed to the skull after clipping. During the surgical treatment, a subdural electrode strip (Wyler, 5-mm diameter; Ad-Tech Medical, Racine, Wisconsin) was placed intraoperatively to permit continuous ECoG monitoring. According to institutional standards for ICP monitoring, either an ICP probe (HD) or an external ventricular drain (EVD) (BE) was placed. After aneurysm treatment, patients were transferred to the ICU. Standard aSAH ICU treatment following national and institutional guidelines [10] was performed. Using the criteria mentioned above, we identified a total of 19 patients (HD *n* = 14 and BE *n* = 5). Demographic data, such as age, sex, neurological examination, aneurysm location, delayed cerebral ischemia (DCI) development, and Glasgow Outcome Scale after 6 months of follow-up, were collected.

### ECoG and ICP Monitoring

ECoG, MAP, and ICP monitoring were performed at the bedside from 3 to 13 days. Continuous ECoG recording was performed from the electrocorticographic six-contact subdural platinum Wyler Strip electrodes. The near-direct current/alternate current ECoG (0.01–45 Hz) was recorded in five active channels, and one contact served as ground. Electrode contacts were connected in a sequential bipolar fashion to an amplifier (AD Instruments, New South Wales, Australia). Data were sampled at 200 Hz and recorded using the Powerlab 16/SP analog-to-digital converter. Registration and analysis of ECoG were done using LabChart v7 (AD Instruments, Bella Vista, Australia). SDs were defined according to the Co-Operative Studies on Brain Injury Depolarizations (COSBID) recommendations [11]. Accordingly, a cluster was defined by the occurrence of at least three SDs occurring within three or fewer consecutive recording hours. Invasive MAP recordings were obtained through femoral or radial artery catheters. In both centers, different methods of ICP monitoring were routinely used. In HD patients, ICP measurements were obtained from a flexible intraparenchymal probe (RAUMEDIC, Helmbrechts, Germany). In BE patients, ICP measurements were collected from an EVD. The EVD management protocol was established according to the ICP measurements. In brief, the drain was kept closed to record ICP measurements, with the transducer mounted 20 cm above Monro’s foramen level. When ICP rose above 20 mm Hg, the drain was opened, and cerebrospinal fluid (CSF) was drained until ICP decreased below 20 mm Hg. The ICP recording was resumed after the EVD was closed to ensure reliable recording data. Analog data of MAP and ICP were sampled at 1/minute in HD patients and 1/second in BE patients from bedside monitors in the ICU monitoring system via TCP/ICP, the Infinity Gateway Software Suite (Dräger Medical Deutschland GmbH, Lübeck, Germany). Monitoring data were stored using ICU Pilot (CMA Microdialysis AB, Solna, Sweden) software.

### Low-Frequency Sample (long) PRx Analysis

Cerebrovascular reactivity using L-PRx was determined as described previously [3, 4]. Briefly, a moving linear (Pearson) correlation coefficient between MAP and ICP was calculated using a minute value in a time window of 20 min with 20 consecutive samples of MAP and ICP. The window was repeated every minute to generate an overlapping index, expressing the correlation for 10 min before and after the desired time point. The value was expressed within a range of − 1 to 1. We assume that a negative value reflects preserved vascular reactivity, whereas a positive L-PRx implies nonreactive vessels. We consider a value greater than 0.2 as a signal of impaired pressure reactivity [3, 4, 12–18]. For every SD event, intervals of 60 min (30 min before and 30 min after the detection of an SD) of L-PRx values were averaged and plotted. Controls were determined by collecting 60-min intervals of random L-PRx values in SD-free periods for more than 2 h. Depending on the type of SD, intervals were categorized as “single SD” or “clustered SDs.”

### Statistical Analysis

Standard descriptive statistics were calculated for L-PRx and SDs. Continuous variables were assessed for normality using histograms and the Kolmogorov–Smirnov and Shapiro–Wilk tests. One-way analysis of variance and Bonferroni post hoc tests were used as parametric statistical methods to compare independent samples. Values of *p* < 0.05 were considered statistically significant. Because all analyses were explorative, no adjustment for multiple tests was applied. Statistical analyses were performed using SPSS v25 (IBM Corp, Armonk, NY).

## Results

The demographic and clinical characteristics of patients are shown in Table 1. A total of 19 patients (mean age 50.4 years, 9 female patients) with aSAH were prospectively monitored for a total of 4,368 h, with a mean duration of 230.4 (STD ± 69.6) recording hours (range: 96–360 h), during which ECoG recordings revealed a total number of 277 SDs. Of these, 184 SDs were single (HD: 121 SDs; BE: 63 SDs), and 93 SDs developed in clusters (HD: 31 SDs; BE: 62 SDs), which occurred in 5 of the 19 patients.

**Table 1:** Clinical and sociodemographic characteristics of patients with aSAH

Patient	Age (years)	Sex (M/F)	WFNS	Aneursym location	DCI	eGOS (6 months)	SD/day	Total SD	Single SD	Clusters of SD	Mean L-PRx
HD-1	55	F	4	Acom	Yes	0	4.6	46	24	22	0.18
HD-2	38	M	4	Acom	Yes	3	1.1	6	6	-	0.10
HD-3	28	F	5	ICA-R	Yes	3	0.4	4	4	-	− 0.04
HD-4	47	M	5	Acom	No	4	0.8	3	3	-	0.26
HD-5	68	F	2	MCA-R	No	6	0	9	9	-	0.15
HD-6	52	F	5	ICA-R	Yes	0	0.6	4	4	-	0.05
HD-7	78	M	5	ICA-R (Pcom)	Yes	3	0.6	5	5	-	0.14
HD-8	46	M	4	MCA-L	No	3	0.4	4	4	-	− 0.20
HD-9	54	F	4	MCA-L	No	2	0.7	5	5	-	0.34
HD-10	52	M	5	Acom	Yes	0	1	4	4	-	0.00
HD-11	61	M	4	Acom	No	3	2.9	35	26	9	0.16
HD-12	48	M	3	ICA-L (Pcom)	No	3	0.8	9	9	-	0.13
HD-13	43	F	3	MCA-L	No	4	1.9	16	16	-	− 0.08
HD-14	46	F	3	MCA-L	No	4	0.7	2	2	-	0.22
BE-1	42	F	4	MCA-R	Probably	5	2.6	22	22	-	0.11
BE-2	53	F	5	MCA-R	Probably	2	1.9	26	1	25	0.15
BE-3	53	M	5	Acom	No	5	1.3	11	11	-	0.24
BE-4	47	M	3	MCA-L	No	4	2	29	23	6	0.09
BE-5	47	F	4	MCA-L	No	3	8.7	37	6	31	0.06

*Acom*, Anterior communicating artery, *aSAH*, Aneurysmal subarachnoid hemorrhage, *BE*, Berlin, *DCI*, Delayed cerebral ischemia, *eGOS*, Extended Glasgow Outcome Scale, *F*, Female, *HD*, Heidelberg, *ICA-L*, Left internal carotid artery, *ICA-R*, Right internal carotid artery, *L-PRx*, Long-pressure reactivity index, *M*, Male, *MCA-L*, Left middle cerebral artery, *MCA-R*, Right middle cerebral artery, *Pcom*, Posterior communicating artery, *SD*, Spreading depolarization, *WFNS*, World Federation of Neurological Surgeons Subarachnoid Hemorrhage Scale

**Fig. 1:**
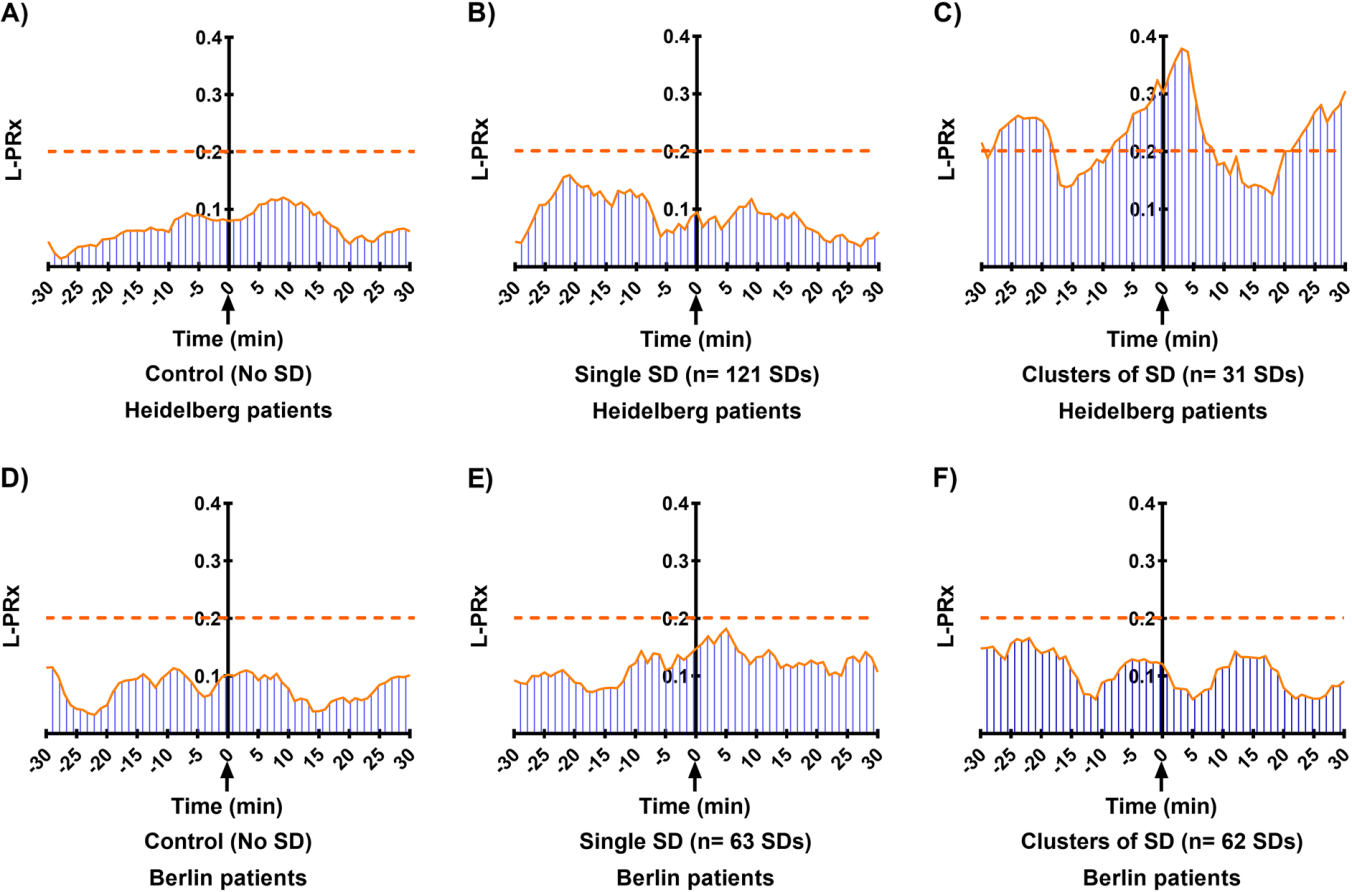
Plots of long-pressure reactivity index (L-PRx) in Heidelberg (**a–c**) and Berlin patients (**d–f**) according to the different time periods are shown. Time windows of 30 min before and after spreading depolarization (SD) detection in electrocorticography (ECoG) were used. Please note the autoregulatory fluctuations of L-PRx during the monitoring time. **c**, Accumulative episodes of L-PRx values greater than 0.2 are exhibited during clusters measured with intracranial pressure (ICP) probes. Please consider that the true origin and time point of SD occurrence might only represent its detection in ECoG in some cases. L-PRx during time periods without SDs (**a** and **d**), during a single SD (**b** and **e**), and during clustered SDs (**c** and **f**). **c**, The time course of L-PRx during SD in clusters shows a peak greater than 0.2, suggesting a loss of autoregulation at the time of SD occurrence. In this group of patients, ICP values were obtained through intraparenchymal ICP probes. A peak L-PRx > 0.3 can be observed 3 min after SD detection

**Fig. 2:**
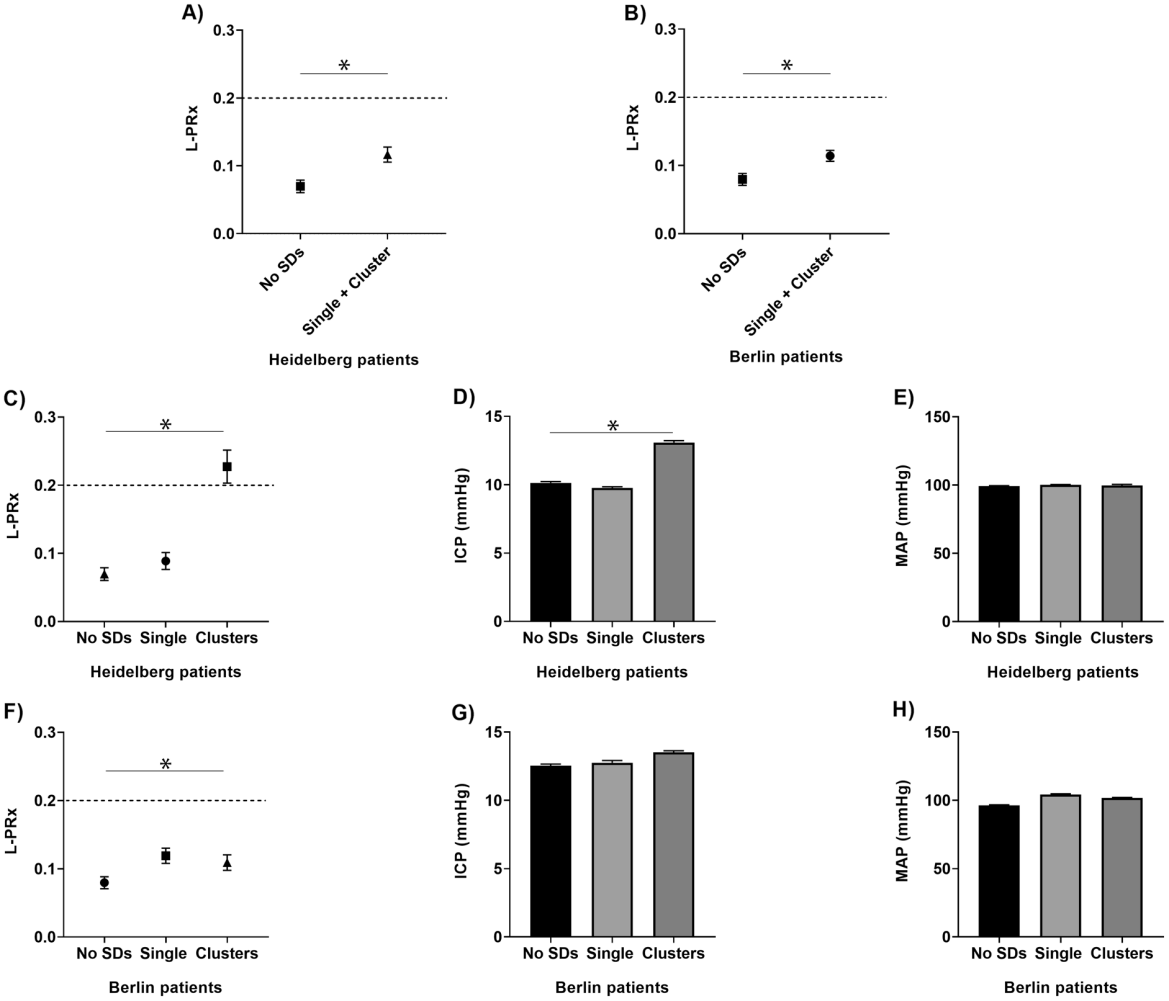
Long-pressure reactivity index (L-PRx) values in patients with aneurysmal subarachnoid hemorrhage (aSAH). **a** and **b**, Box plots show autoregulation differences between periods with and without spreading depolarizations (SDs) in Heidelberg (HD) and Berlin (BE) patients. In both groups, HD and BE, significant increases of L-PRx values > 0.1 during SDs were detected (*p* < 0.001). **c–e**, L-PRx, intracranial pressure (ICP), and mean arterial pressure (MAP) values of HD patients are shown according to the different time periods. **c**, A loss of autoregulation (L-PRx > 0.2) was detected during clustered SDs (*p* < 0.001). **d**, Mean ICP values according to the different time periods are shown. ICP values were obtained through an intraparenchymal probe. Significantly higher ICP values were detected during SD in clusters (*p* < 0.001). **e**, MAP values of HD patients during the different time periods are shown. No difference was found between periods (*p* > 0.05). **f–h**, L-PRx, ICP, and MAP values of BE patients are shown according to the different periods. **f**, Mean L-PRx values during single and clustered SDs remained above 0.1. These values were still higher than those in SD-free periods. **g** and **h**, No differences between time periods in mean ICP and MAP values were found in BE patients (*p* > 0.05). Data are expressed as mean and 95% confidence interval

A schematic representation of L-PRx fluctuations from HD and BE patients for the different periods are shown in Fig. 1. In HD and BE patients, significant increases in L-PRx were found during the development of SDs in comparison with control periods. In HD patients, L-PRx values were 0.12 (95% confidence interval [CI] 0.11–0.13) during SDs and 0.07 (95% CI 0.06–0.08) during control periods (*p* < 0.001). Similarly, in BE patients, L-PRx values were 0.11 (95% CI 0.11–0.12) during SDs and 0.08 (95% CI 0.07–0.09) in control periods (*p* < 0.001) (Figs. 2a, b). In a more detailed analysis according to the type of ICP measurement method, CA changes recorded through an intraparenchymal probe (HD patients) revealed that clustered SD periods were characterized by signs of more severe CA impairment (L-PRx > 0.2) (Fig. 1c). In contrast, L-PRx values during single SD and control periods consistently remained < 0.2 in HD patients, indicating intact CA, compared with MAP/ICP recording periods during clustered SDs (Figs. 2a, b). In HD patients, a significant L-PRx difference (*F* = 69.0, degrees of freedom [df] 2, *p* < 0.001) between periods of clustered SDs (L-PRx: 0.23, 95% CI 0.20–0.25), single SD (L-PRx: 0.09, 95% CI 0.08–0.10) and control periods (L-PRx: 0.07, 95% CI 0.06–0.08) was found (Fig. 2c). In this group, significant increases in ICP values during clustered SDs (mean 13.1 mm Hg, 95% CI 13.0–13.2) compared with single SD (mean 9.8 mm Hg, 95% CI 9.7–9.9) and control periods (mean 10.1 mm Hg, 95% CI 10.1–10.2) were additionally detected (*F* = 465.2, 2 df, *p* < 0.001) (Fig. 2d). No significant differences in MAP between the different periods were found (Fig. 2e).

In BE patients, no loss of autoregulation was found when using ICP changes detected in EVD during clustered SD periods. Mean L-PRx values during SD in clusters (L-PRx: 0.11, 95% CI 0.10–0.12) remained under the threshold of 0.2. However, as mentioned above, these values were still higher than those observed in the SD-free periods (L-PRx: 0.08, 95% CI 0.07–0.09), and a significant difference between all the periods (no SD, single SD, and clusters) was found (*F* = 17.1, 2 df, *p* < 0.001) (Fig. 2f). In BE patients, no significant differences in ICP or MAP between the different periods were found (Figs. 2g, h).

## Discussion

The present work shows the general feasibility of performing CA assessment based on cerebrovascular pressure reactivity during periods surrounding SD development. Consistently, higher L-PRx values during SD periods than SD-free periods suggested a higher likelihood of CA disturbance. In particular, an evident CA disruption with L-PRx values > 0.2 could be detected during the development of clustered SDs when using intraparenchymal ICP measurements. This could at least partially explain why the occurrence of clustered SDs might be associated with a higher likelihood of suffering secondary insults after aSAH. These findings need to be confirmed in a larger prospective patient cohort.

In the healthy brain, increases in MAP will induce cerebral vasoconstriction, with a subsequent decrease in ICP and cerebral blood volume. In cases of impaired cerebrovascular reactivity, increases in MAP will lead to an increase in ICP due to a passive response of the cerebral resistance vessels [1–4]. By this background, L-PRx permits a continuous estimation of cerebrovascular reactivity as a surrogate marker for CA, thereby reflecting the capacity of cerebral resistance vessels to modify their diameter in response to perfusion pressure changes [2–4]. After aSAH, CA is often compromised [19], and the normal hemodynamic response to SD can be inverted [20]. Different hemodynamic responses with different overlapping vascular components have been identified in the gyrencephalic brain [21–23]. Thus, progressively prolonged SD-induced spreading ischemia with intense vasoconstriction and transition from clustered SDs to a negative ultraslow potential were found when optoelectrodes were located directly over a newly developing delayed cerebral infarct detected by serial neuroimaging after SAH [24]. If it is associated with SD in clusters and ischemic hemodynamic responses, CA impairment could possibly be a predictor of these pathophysiological changes.

In the present study, we cannot directly link a spatial–temporal association between SDs and L-PRx impairment. The limited spatial resolution of the six-contact subdural ECoG electrode does not allow a clear identification of the true origin and time point onset of SDs compared to the more global reflection of CA based on MAP and ICP pressure reactivity. Even more, it is well known that SDs move in irregular patterns [25, 26]. As another limitation, our sample size is small. Only two HD patients and three BE patients had both single and clustered SDs. In addition, differences were more considerable when using intraparenchymal than intraventricular ICP measurements. Thus, our observation should be taken as preliminary evidence that there may be a link between SD in clusters and loss of autoregulation. Similar findings supporting this hypothesis have been published by Owen et al. in a recent study assessing the relationship between different CA indices and the relation between SDs and outcomes [27]. They calculated, among other indices, PRx by using an intraparenchymal probe and an EVD for ICP monitoring simultaneously. They observed that in contrast to the PRx obtained from an EVD, the PRx obtained from an intraparenchymal probe performed flawlessly in differentiating between good and poor outcomes and was significantly associated with SD incidence. They proposed that optimizing CA may lead to decreased SD incidence, improving patient outcomes.

A direct causal effect of SD on CA impairment has not yet been established. Experimental data over the last 28 years using various animal models, including rats, rabbits, and cats, have concluded that the period following SD is characterized by impaired cerebrovascular reactivity to changes in partial pressure of carbon dioxide (pCO2) and an impaired neurovascular response to functional activation [28]. This is even true for SDs with a normal hemodynamic response. The normal hemodynamic response to SD must be distinguished from the inverse hemodynamic response, which occurs only in severe neurovascular unit disorders and can cause cerebral infarcts [8, 20]. In the clinical setting, an inverse hemodynamic response to SD in patients with TBI was related to a progressive deterioration in autoregulatory function [9]. Several human and experimental studies indicate that after TBI, the CA is heterogeneously impaired through various cellular mechanisms that affect myogenic tone [29], including the production of peroxynitrite (ONOO^−^) (which impairs myogenic dilation) [30] and excessive production of nitric oxide (NO) (which affects myogenic constriction) [31]. SDs occur in about 60% of patients with surgical TBI [29, 32]. Diverse experimental analyses have acknowledged numerous molecular pathways activated in the acute and subacute stages of SAH that could considerably contribute to CA disturbances through the impairment of vasomotor and vasodilatory responses (after an increased formation of peroxides and a reduced production of prostacyclin, respectively) as well as due to secondary brain injury (such as blood–brain barrier disruption) that causes endothelial dysfunction [33]. Thus, although the direct mechanisms in which SD impairs CA are not known, it would result from a multifactorial process that follows SAH. Hinzman et al. [9], for example, proposed that an impairment of CA through SD could be possibly explained through the metabolic hypothesis of autoregulation, in which an energy supply–demand mismatch would cause the release of chemical factors that alter the vascular tone, an effect that has already been explored in experimental models of TBI [29, 34]. Finally, DCI is a complex mechanism of delayed injury in SAH with multifactorial pathophysiology [35]. A direct effect of SD on the development of DCI after SAH could be possible through three potential mechanisms: (1) An increasing hypoxic response to SD [36, 37] that would contribute to cortical microvasospasm; (2) An inverse neurovascular coupling to SD as a result of decreased NO and elevated extracellular potassium (K^+^), which would promote a shift to an inverse hemodynamic response [9, 29, 36, 37]; and (3) An impaired CA after SD, which would potentially contribute to the development of an inverse hemodynamic response [38, 39]. Although not mechanistically explained yet, various studies in SAH have already proven a strong correlation between SD, particularly in clusters, impairment of CA, and DCI development [19, 38, 40–43]. Additional research would be necessary to clarify an association between all these variables.

Higher ICP values were associated with periods of clustered SDs compared with periods with a single SD or control periods when using intraparenchymal ICP probes. Recently, it has been suggested that mild and brief ICP spikes may be capable of triggering SDs [44]. A similar observation has been previously reported in a small study of patients with aSAH, in which higher ICP values were detected during clusters of recurrent SDs than during isolated SDs [45]. Also, a small but significant increase of ICP related to SD occurrence was found in patients with malignant hemispheric stroke. ICP started rising 1 h before SDs and remained elevated until 2 h after the event [46]. In patients with TBI, ICP values during SDs did not differ from those obtained throughout ECoG monitoring. However, ICPs were significantly higher in patients with SDs than those without [47].

Additionally, the fact that L-PRx was more impaired in cases of higher ICP with potentially lower cerebral perfusion pressure appears to agree with our observation in patients with malignant hemispheric stroke, in which CA impairment followed a perfusion-dependent pattern, with more significant CA impairment at low cerebral perfusion levels [48]. Moreover, a pressure reactivity index such as L-PRx may also reflect a change in brain elasticity, which changes during SDs [49], and less adaptability to changes in MAP, produced by brain cytotoxic edema as a consequence of neuronal swelling [6] and dendritic beading that occurs during SDs [50]. However, this hypothesis needs to be tested in further studies. The pharmacological blocking of SDs could help answer those questions [21, 51, 52].

Here we found that L-PRx values during clustered SDs exceeded the cutoff value of 0.2, whereas L-PRx values of the combined single and clustered SDs did not. However, the presence of isolated SD still statistically differed from SD-free periods. In this sense, the elevation of the L-PRx value could still be meaningful, although it remained below the threshold value. Although some reports defined 0.3 as a cutoff value for autoregulatory failure, there is evidence from different authors during the last 25 years that support a cutoff value of 0.2 in indices such as PRx or L-PRx being associated with a significant disturbance of pressure reactivity and an increase in the mortality rate in different pathological conditions, such as TBI and intracerebral hemorrhage [3, 4, 12–18]. PRx values below 0.2 for a 2-h period have been associated with poor prognosis in patients with brain injury [13, 53]. Specific differences may arise in the measurement of ICP by using EVD and intraparenchymal devices. In this study, we presented data from two different cohorts that might differ slightly from their management protocols. Therefore, the true impact of SD on CA measured by either an EVD or an intraparenchymal probe for ICP monitoring should be taken cautiously. For example, the presence of an impaired CA during SD in clusters in HD patients, but not in BE patients, could reflect the effect of continuous measurements with an intraparenchymal probe instead of intermittent measurements through an EVD. In this regard, although EVD is the gold standard for ICP measurement [54], there is an ongoing debate regarding the choice of a monitoring device in patients with aSAH because it seems that EVD is associated with an increased risk of aneurysmal rebleeding, intracerebral hemorrhage, and infection, and guidelines to standardize indications for ICP measurement and therapeutic targets are scarce [55]. Intraparenchymal devices, on the contrary, are relatively easy to place and offer a lower rate of hemorrhage and infection, yet unlike EVD, they cannot be calibrated after placement [54, 55]. On the other hand, when using EVD, changes in intracranial elastance can occur as a result of therapeutic interventions during neurocritical care, such as CSF drainage [56], limiting the continuity of ICP data acquisition, which could impact CA measurements. Further evidence suggests that ICP monitoring by an open EVD may lead to a less reliable continuous assessment of CA [57].

## Conclusions

Multimodal neuromonitoring for simultaneous assessment of cerebrovascular pressure reactivity, as measured by L-PRx using 20-min averages of MAP and ICP, together with ECoG recordings, allows for the evaluation of CA during periods surrounding SD development. SD occurrence was associated with significant increases in L-PRx values indicative of CA disturbances. An impaired CA was found during SD in clusters when using an intraparenchymal probe. The reason for this is unknown but could indicate a slight difference in continuous data acquisition between both methods. Nevertheless, this should be taken with caution because of the number of study participants in our study. This preliminary study validates the use of cerebrovascular reactivity indices to evaluate CA disturbances during SDs. Our results warrant further investigation in larger prospective patient cohorts.

## Supplementary Information

Below is the link to the electronic supplementary material.

Supplementary file1 (JPG 709 KB)
