# Supplementary figures and images for: Cerebrovascular Pressure Reactivity According to Long-Pressure Reactivity Index During Spreading Depolarizations in Aneurysmal Subarachnoid Hemorrhage
Source: Neurocrit Care. 2023 Jan 25;39(1):135–44. doi: 10.1007/s12028-022-01669-y (PMC10499750; doi:10.1007/s12028-022-01669-y)

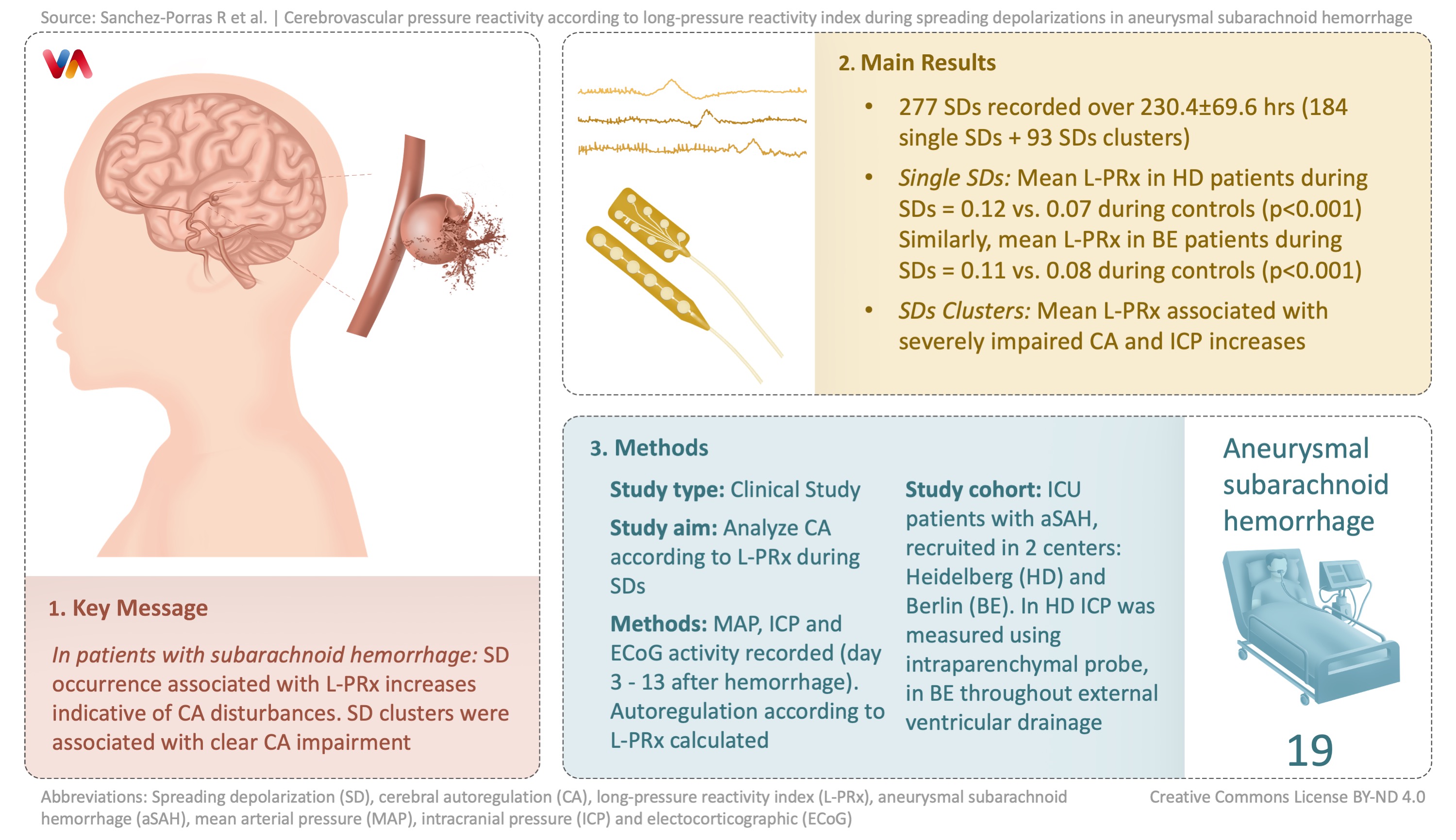

Supplement: Supplementary file 1 — Supplementary file1 (JPG 709 KB) [file 12028_2022_1669_MOESM1_ESM.jpg]
